# T cell immune quiescence as a contributor to resistance to infection among HIV Exposed Seronegative (HESN) commercial sex workers from Nairobi, Kenya

**DOI:** 10.1186/1742-4690-9-S2-P205

**Published:** 2012-09-13

**Authors:** KR Fowke, TB Ball, J Kimani, FA Plummer

**Affiliations:** 1 University of Manitoba, Winnipeg, Canada; 2 Public Health Agency of Canada, Winnipeg, Canada; 3 Kenyan AIDS Control Program, Nairobi, Kenya

## Background

Participants from the Majengo Commerical Sex Worker Cohort in Nairobi, Kenya have been intensely exposed to HIV for 7-20 years of follow-up, and yet have remained uninfected. Since activated CD4+ T cells are much more susceptible to HIV infection and have been suggested to form the initial focus of mucosal infection, we have conducted a number of studies to characterize the T cell phenotype in the mucosal and systemic compartments of these HIV exposed seronegative women (HESN).

## Methods

Representative sampling (n=~30) of HESN women and a similar sized control group of newly enrolled commercial sex workers were compared. Gene expression analysis, immune phenotyping, and in vitro HIV infection assays were performed on peripheral blood mononuclear cells. Mucosal assessment of the female genital tract (FGT) included proteomic analysis by mass spectrometry, flow cytometry and cytokine/chemokines determinations by bead arrays.

## Results

Gene expression analysis revealed the HESN women showed reduced gene levels for pathways involved in T cell receptor activation and HIV host dependent factors. Systemic CD4+ T cells showed lower levels of immune activation (CD69) and higher levels of regulatory T cells (T regs). Infection frequency, the number of infected replicate wells, was lower in the HESN women and correlated with ex vivo assessment of reduced T cell activation and elevated T reg levels. Mucosal assessment by proteomics showed higher levels of anti-inflammatory serine proteases and lower levels of the chemokines IP-10 and MIG, which functions are to recruit activated T cells into the mucosal environment.

## Conclusion

Together, these data suggest that the HESN women of the Majengo Cohort display a T cell Immune Quiescent phenotype that is characterized by fewer activated T cells, more regulatory T cells and a mucosal environment that favours quiescent cells. The result is an environment that is not favorable for the establishment of HIV infection.

